# Supplementary material for: The Impact of Having One Parent Absent on Children’ Food Consumption and Nutrition in China
Source: Nutrients. 2019 Dec 17;11(12):3077. doi: 10.3390/nu11123077 (PMC6950458; doi:10.3390/nu11123077)
Supplement: Supplementary file 1 [file nutrients-11-03077-s001.pdf]

Supplementary file

Figure S.1 Flow chart

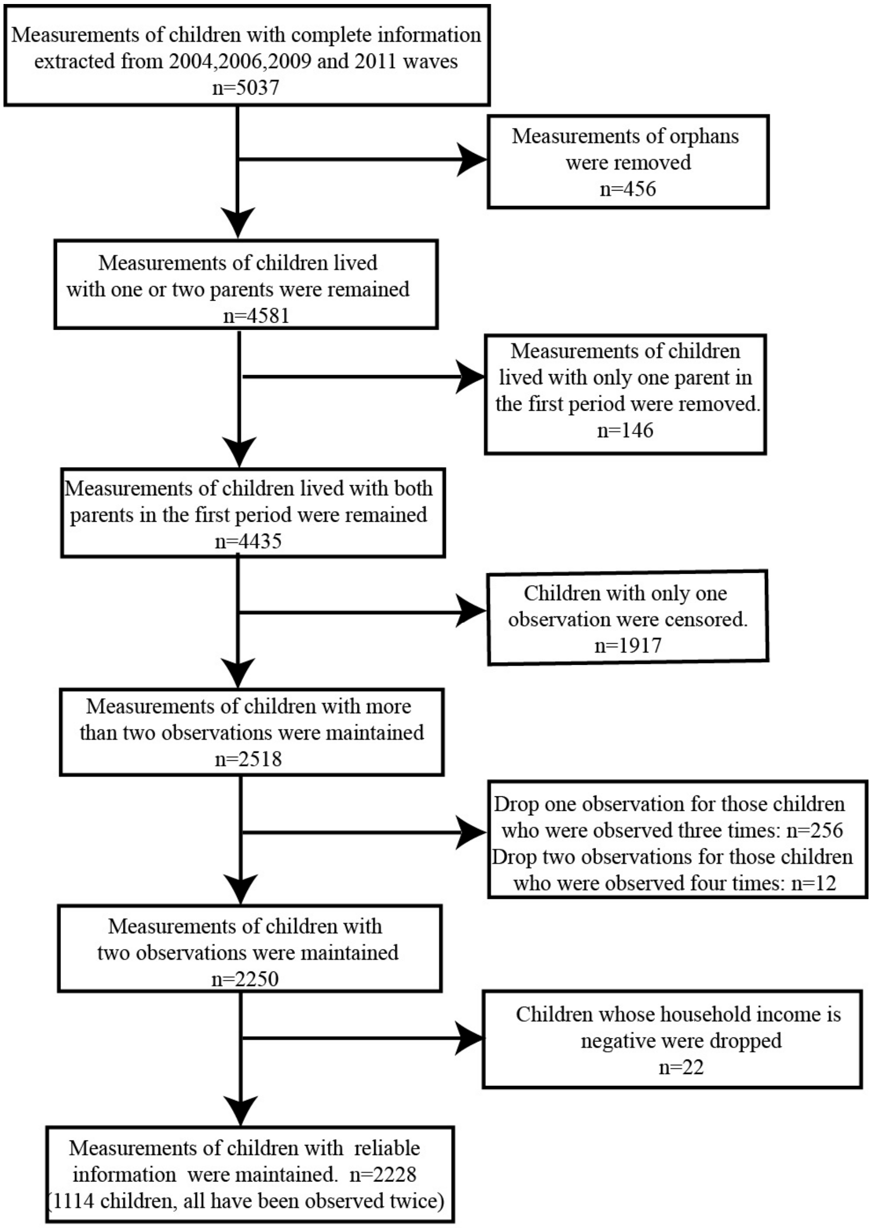

Table S.1 Balance test for co-variants used in matching

Matching variables	Single-parent v.s. Dual-parent				Single-mother v.s. Dual-parent				Single-father v.s. Dual-parent			
	Dual-	Single-	Diff.	p	Dual-	Single-	Diff.	p	Dual-	Single-	Diff.	p
ln(income)	9.25	9.24	-0.01	0.95	9.23	9.07	-0.16	0.16	9.65	9.63	-0.02	0.76
Household size	5.40	5.49	0.08	0.52	5.03	5.13	0.10	0.41	6.28	6.27	-0.01	0.96
Children share	0.37	0.37	0.00	0.62	0.37	0.37	0.01	0.59	0.36	0.37	0.01	0.27
Children-age	7.47	7.52	0.05	0.82	7.44	7.28	-0.16	0.49	7.72	7.93	0.21	0.42
Children-gender	0.49	0.49	0.00	0.91	0.49	0.51	0.01	0.66	0.47	0.46	-0.01	0.88
Household head-age	46.55	47.30	0.76	0.42	45.22	45.18	-0.05	0.96	51.01	51.76	0.74	0.50
Household head-activity	3.07	3.06	-0.01	0.88	3.06	3.06	0.01	0.95	3.02	3.05	0.03	0.69
Household head-gender	0.40	0.41	0.02	0.57	0.40	0.42	0.02	0.45	0.36	0.37	0.01	0.78
Household head-education	1.40	1.34	-0.06	0.36	1.47	1.44	-0.03	0.64	1.26	1.15	-0.11	0.14
Urban dummy	0.87	0.88	0.01	0.54	0.86	0.87	0.00	0.88	0.89	0.90	0.01	0.60
Time gap	2.78	2.80	0.02	0.81	2.81	2.84	0.02	0.80	2.75	2.71	-0.05	0.61
Provincial dummy 1	0.04	0.03	-0.01	0.46	0.04	0.04	0.00	0.91	0.04	0.02	-0.02	0.20
Provincial dummy 2	0.09	0.09	0.00	0.81	0.12	0.11	0.00	0.91	0.03	0.02	-0.01	0.43
Provincial dummy 3	0.06	0.05	-0.01	0.74	0.04	0.04	0.00	0.83	0.08	0.07	0.00	0.83
Provincial dummy 4	0.11	0.11	0.00	0.96	0.14	0.14	0.00	1.00	0.04	0.02	-0.01	0.31
Provincial dummy 5	0.07	0.07	0.00	0.95	0.09	0.08	0.00	0.83	0.05	0.05	0.00	0.89
Provincial dummy 6	0.06	0.06	0.00	0.82	0.08	0.07	-0.01	0.74	0.03	0.02	-0.01	0.50
Provincial dummy 7	0.30	0.32	0.02	0.59	0.28	0.31	0.03	0.30	0.29	0.34	0.05	0.15
Provincial dummy 8	0.20	0.20	0.01	0.75	0.11	0.10	-0.01	0.65	0.40	0.42	0.02	0.63

Notes: *, **, *** refer statistically significant at 10%, 5%, and 1%. Values in brackets are standard deviation/error. Definitions of activity and education can be found in S table 1.

Table S.2 Comparison of food consumption and nutrition indicator after matching

	Dietary indicator	Initial period			Following up period		
		Dual-	Single-	Diff.	Dual-	Single-	Diff.
Food consumption	Cereals (g)	253.83	267.10	13.27	293.16	301.13	7.97
	Vegetables (g)	177.00	189.01	12.02	179.96	199.51	19.55**
	Meat and poultry (g)	63.07	55.51	-7.55**	67.30	63.39	-3.90
	Aquatic products (g)	16.35	9.57	-6.78***	13.69	8.15	-5.54***
	Eggs (g)	18.93	16.31	-2.62	20.15	22.55	2.40
	Dairy products (g)	10.72	9.04	-1.69	8.53	4.88	-3.65
	Fruits (g)	36.10	25.80	-10.31*	58.63	61.87	3.24
Nutrition intake	Calorie (kilocalorie)	1118.46	1129.51	11.05	1179.71	1239.81	60.10*
	Carbohydrate (g)	192.62	200.93	8.32	203.68	219.85	16.17***
	Fat (g)	23.63	21.26	-2.37**	24.68	24.47	-0.21
	Protein (g)	39.39	39.49	0.10	41.99	41.59	-0.40
	Vitamin A (μg)	265.12	289.44	24.32	303.41	307.84	4.44
	Vitamin B (mg)	9.61	9.71	0.11	10.35	10.56	0.21
	Vitamin C (mg)	45.53	45.53	0.00	47.52	52.52	5.00**
	Vitamin E (mg)	6.43	6.33	-0.10	7.05	7.11	0.06
	Calcium (mg)	215.22	219.98	4.76	227.51	237.38	9.87
	Iron (mg)	11.49	12.19	0.70	12.82	13.23	0.41
	Zinc (mg)	6.47	6.57	0.10	6.97	7.22	0.25
	Selenium (μg)	25.42	23.68	-1.74	27.73	27.72	-0.01

Notes: *, **, *** refer statistically significant at 10%, 5%, and 1%. Vitamin A is measured by Retinol Equivalent.

Table S.3 Comparison of food consumption and nutrition indicator after matching

Family type		Single-mother v.s. Dual-parent						Single-father v.s. Dual-parent					
Food and nutrition		Initial period			Following up period			Initial period			Following up period		
		Dual-	Single-	Dual-	Single-	Dual-	Single-	Dual-	Single-	Dual-	Single-	Dual-	Single-
Food consumption	Cereals (g)	253.03	280.73	27.70***	288.40	314.11	25.70***	257.01	233.84	23.17**	313.36	261.80	-51.57***
	Vegetables (g)	176.55	197.01	20.47**	177.32	193.52	16.20*	175.85	166.22	-9.63	196.42	202.44	6.02
	Meat and poultry (g)	62.16	61.03	-1.23	66.65	65.26	-1.39	62.41	42.05	-20.36***	66.88	58.25	-8.63**
	Aquatic products (g)	17.92	11.23	-6.69***	15.35	8.75	-6.61***	13.48	5.65	-7.83***	9.77	6.75	-3.02*
	Eggs (g)	21.23	16.51	-4.72***	21.89	24.05	2.16	15.31	15.92	0.61	17.17	19.82	2.65
	Dairy products (g)	12.73	9.83	-2.90	10.13	3.78	-6.35**	5.76	11.23	5.46*	6.73	7.48	0.75
	Fruits (g)	41.82	28.84	-12.98**	59.94	64.32	4.38	27.01	18.70	-8.31	58.77	53.64	-5.13
Nutrition intake	Calorie (kilocalorie)	1122.13	1176.74	54.62	1186.51	1276.24	89.73***	1098.24	1013.87	-84.37**	1239.03	1134.88	-104.15***
	Carbohydrate (g)	192.45	207.88	15.43**	203.76	228.06	24.30***	191.60	183.50	-8.10	220.10	198.49	-21.61***
	Fat (g)	23.95	22.69	-1.26	25.25	24.17	-1.08	22.21	17.95	-4.26***	23.97	24.04	0.07
	Protein (g)	39.88	41.66	1.78	42.37	43.26	0.90	38.25	34.20	-4.05***	42.84	37.27	-5.57***
	Vitamin A (μg)	272.29	310.78	38.49	294.41	324.85	30.44	250.94	238.89	-12.05	307.96	262.45	-45.51**
	Vitamin B (mg)	9.63	10.36	0.73**	10.24	10.51	0.27	9.49	8.11	-1.38***	10.82	10.32	-0.50
	Vitamin C (mg)	45.46	47.55	2.09	46.11	49.44	3.33	46.10	39.98	-6.12**	52.45	58.26	5.81*
	Vitamin E (mg)	6.60	6.67	0.07	7.19	7.33	0.15	6.02	5.49	-0.53	6.99	6.37	-0.61
	Calcium (mg)	221.52	228.87	7.35	228.80	230.26	1.46	205.22	201.14	-4.08	225.99	248.60	22.62*
	Iron (mg)	11.59	12.96	1.38***	12.90	13.72	0.82	11.28	10.29	-0.99**	13.32	11.87	-1.45***
	Zinc (mg)	6.48	6.83	0.35*	6.93	7.33	0.40*	6.42	5.92	-0.50**	7.43	6.79	-0.64***
	Selenium (μg)	26.18	25.53	-0.65	28.40	29.85	1.45	24.92	19.35	-4.94***	29.13	22.52	-6.61***

Notes: *, **, *** refer statistically significant at 10%, 5%, and 1%. Vitamin A is measured by Retinol Equivalent.

Table S.4 Model specification test in estimating income elasticity

	Family type	Single-parent			Single-mother		Single-father		Hausman
	Models	FE	RE	Hausman	FE	RE	FE	RE	
Food consumption	Cereals	-4.95	-2.99	38.57***	-4.97	-1.05	-4.81	-6.95	40.12***
	Vegetables	0.41	-1.06	46.27***	2.22	-0.55	-11.25	-2.08	47.61***
	Meat and poultry	6.73	2.89	58.32***	7.42	3.43	2.26	1.75	59.48***
	Aquatic products	-1.13	0.30	24.22***	-0.90	0.45	-2.56	0.01	24.59***
	Eggs	-0.42	-0.24	22.78***	-0.32	-0.17	-1.02	-0.39	22.88***
	Dairy products	-1.11	1.14	29.66***	-1.36	0.98	0.51	1.50	29.89***
	Fruits	-2.31	3.71	28.08***	0.66	4.13	-21.52	2.89	29.56***
Nutrition intake	Calorie	1.02	4.39	61.83***	5.76	10.98	-29.55	-8.65	64.58***
	Carbohydrate	-4.06	-1.05	66.92***	-4.24	0.13	-2.87	-3.38	70.50***
	Fat	1.77	0.76	46.99***	2.22	0.87	-1.14	0.56	48.71***
	Protein	-0.24	0.31	58.87***	0.12	0.60	-2.57	-0.24	61.81***
	Vitamin A	27.87	3.42	9.62	37.48	6.52	-34.25	-2.73	10.38
	Vitamin B	0.07	0.13	46.65***	0.12	0.18	-0.26	0.03	48.28***
	Vitamin C	-0.40	-0.03	20.06***	-0.21	0.01	-1.63	-0.13	20.28***
	Vitamin E	-0.45	0.13	20.13***	-0.33	0.17	-1.24	0.04	21.28***
	Calcium	-2.72	3.09	32.57***	4.68	3.51	-50.58	2.21	35.95***
	Iron	-0.02	0.13	15.21**	0.03	0.23	-0.34	-0.06	15.99**
	Zinc	0.11	0.06	55.25***	0.18	0.10	-0.32	0.00	57.86***
	Selenium	0.53	0.21	20.91***	0.82	0.48	-1.34	-0.32	21.41***

Notes: *, **, *** refer statistically significant at 10%, 5%, and 1%. FE and RE refer to fixed-effect model and random-effect model, which are two econometric models widely used for short-time panel data. Hausman refers to the model comparison test between FE and RE, where rejection of null hypothesis indicates FE is more preferred.

Table S.5 Initial value of dietary indicators and income elasticity

Family type		Initial value			Income elasticity		
		Single-	Single-mother	Single-father	Single-	Single-mother	Single-father
Food consumption	Cereals (g)	264.89	280.73	229.16	-0.019	-0.018	-0.021
	Vegetables (g)	187.07	197.01	164.65	0.002	0.011	-0.068
	Meat and poultry (g)	54.72	61.03	40.48	0.123	0.122	0.056
	Aquatic products (g)	9.44	11.23	5.39	-0.120	-0.080	-0.475
	Eggs (g)	16.22	16.51	15.57	-0.026	-0.019	-0.066
	Dairy products (g)	10.10	9.83	10.71	-0.110	-0.138	0.048
	Fruits (g)	25.45	28.84	17.83	-0.091	0.023	-1.207
Nutrition intake	Calorie (kilocalorie)	1119.50	1176.74	990.36	0.001	0.005	-0.030
	Carbohydrate (g)	199.25	207.88	179.77	-0.020	-0.020	-0.016
	Fat (g)	21.04	22.69	17.30	0.084	0.098	-0.066
	Protein (g)	39.12	41.66	33.41	-0.006	0.003	-0.077
	Vitamin A (μg)	286.13	310.78	230.53	0.012	0.021	-0.012
	Vitamin B (mg)	9.61	10.36	7.92	0.007	0.012	-0.033
	Vitamin C (mg)	45.08	47.55	39.53	-0.009	-0.004	-0.041
	Vitamin E (mg)	6.27	6.67	5.37	-0.072	-0.049	-0.231
	Calcium (mg)	218.93	228.87	196.52	-0.012	0.020	-0.257
	Iron (mg)	12.07	12.96	10.06	-0.002	0.002	-0.034
	Zinc (mg)	6.51	6.83	5.79	0.017	0.026	-0.055
	Selenium (μg)	23.50	25.53	18.94	0.023	0.032	-0.071

Notes: Vitamin A is measured by Retinol Equivalent.

Table S.6 Income changes between two periods and ATT of income

Income	Single-parent	Single-mother	Single-father	Dual-parents
First period	17769	16630	20338	22545
Second period	20538	19888	22003	32378
Changes	2769	3258	1665	9833
ATT	-6610	-5637	-8467	

Table S.7 ATT of single-parent children using various kernel density functions

	Dietary indicator	Single-parent vs. Dual-parent				Single-mother v.s. Dual-parent				Single-mother v.s. Dual-parent			
		epanechnikov	biweight	uniform	tricube	epanechnikov	biweight	uniform	tricube	epanechnikov	biweight	uniform	tricube
Food consumption	Cereals (g)	-5.30	-6.27	-3.82	-4.06	-1.99	-2.18	-1.37	-1.64	-28.40**	-27.01*	-28.58**	-29.89**
	Vegetables (g)	7.54	7.28	7.40	7.66	-4.27	-4.44	-3.79	-4.06	15.65	16.87	14.75	14.62
	Meat and poultry (g)	3.65	3.45	3.99	3.92	-0.27	-0.45	0.25	-0.00	11.73**	11.62**	12.27**	11.74**
	Aquatic products (g)	1.24	1.14	1.34	1.37	0.08	0.28	-0.17	-0.19	4.81**	4.49*	5.34**	5.12**
	Eggs (g)	5.02**	4.98**	4.99**	5.05**	6.89***	6.95***	6.78***	6.81***	2.04	1.91	2.10	2.16
	Dairy products (g)	-1.97	-2.01	-1.84	-1.89	-3.44	-3.46	-3.45	-3.39	-4.72	-4.87	-4.42	-4.50
	Fruits (g)	13.54	13.04	14.65*	14.20*	17.36*	17.27*	17.31*	17.42*	3.18	3.29	3.26	2.88
Nutrition intake	Calorie (kilocalorie)	49.05	47.35	49.79	51.12	35.11	34.05	37.26	36.51	-19.78	-15.52	-21.87	-24.69
	Carbohydrate (g)	7.85	7.59	7.90	8.15	8.87	8.75	9.13	9.04	-13.51	-12.72	-13.39	-14.18
	Fat (g)	2.17	2.11	2.22	2.34	0.18	0.12	0.27	0.24	4.32***	4.46***	3.98**	4.07**
	Protein (g)	-0.50	-0.55	-0.47	-0.43	-0.88	-0.90	-0.79	-0.84	-1.52	-1.52	-1.47	-1.58
	Vitamin A (μg)	-19.89	-20.32	-17.50	-19.15	-8.04	-6.94	-8.96	-9.10	-33.45	-31.66	-33.07	-34.43
	Vitamin B (mg)	0.10	0.09	0.13	0.13	-0.46	-0.46	-0.44	-0.45	0.88*	0.90*	0.88*	0.85*
	Vitamin C (mg)	5.00	4.88	5.20	5.14	1.24	1.19	1.32	1.30	11.93***	12.07***	11.77***	11.78***
	Vitamin E (mg)	0.16	0.14	0.17	0.17	0.07	0.07	0.09	0.09	-0.08	-0.06	-0.16	-0.14
	Calcium (mg)	5.11	4.71	5.79	5.68	-5.89	-6.06	-5.55	-5.59	26.70	26.13	26.65	26.87
	Iron (mg)	-0.29	-0.28	-0.30	-0.30	-0.56	-0.56	-0.54	-0.55	-0.46	-0.47	-0.37	-0.43
	Zinc (mg)	0.16	0.15	0.16	0.17	0.06	0.05	0.07	0.06	-0.14	-0.14	-0.12	-0.15
	Selenium (μg)	1.73	1.64	1.85	1.84	2.10	2.13	2.08	2.06	-1.67	-1.40	-1.65	-1.86

Notes: *, **, *** refer statistically significant at 10%, 5%, and 1%. Vitamin A is measured by Retinol Equivalent. All results are estimated in Stata using the command “diff”.

Table S.8 Income effect and compensation effect-results from fixed income elasticity model

	Family type	Single-parent			Single-mother			Single-father		
		ATT	Income effect	Compensation effect	ATT	Income effect	Compensation effect	ATT	Income effect	Compensation effect
Food consumption	Cereals (g)	-5.30	3.94	-9.24	-1.99	3.81	-5.80	-28.40	8.59	-36.99
	Vegetables (g)	7.54	1.67	5.87	-4.27	0.33	-4.60	15.65	9.94	5.71
	Meat and poultry (g)	3.65	-2.48	6.13	-0.27	-2.03	1.76	11.73	-4.72	16.45
	Aquatic products (g)	1.24	0.27	0.97	0.08	0.36	-0.28	4.81	-0.05	4.86
	Eggs (g)	5.02	0.15	4.87	6.88	0.31	6.57	2.04	-1.15	3.19
	Dairy products (g)	-1.97	-0.02	-1.95	-3.44	-0.02	-3.42	-4.72	-0.01	-4.71
	Fruits (g)	13.54	1.31	12.23	17.36	0.45	16.91	3.18	5.43	-2.25
Nutrition intake	Calorie (kilocalorie)	49.05	0.00	49.05	35.11	-5.19	40.30	-19.78	35.46	-55.24
	Carbohydrate (g)	7.85	1.85	6.00	8.87	1.27	7.60	-13.51	5.24	-18.75
	Fat (g)	2.17	-0.59	2.76	0.18	-0.78	0.96	4.32	0.70	3.62
	Protein (g)	-0.50	0.04	-0.54	-0.88	-0.20	-0.68	-1.52	1.59	-3.11
	Vitamin A (μg)	-19.89	-5.22	-14.67	-8.04	-8.22	0.18	-33.45	13.82	-47.27
	Vitamin B (mg)	0.10	0.00	0.10	-0.46	-0.05	-0.41	0.88	0.29	0.59
	Vitamin C (mg)	5.00	1.01	3.99	1.24	0.92	0.32	11.93	1.32	10.61
	Vitamin E (mg)	0.16	0.14	0.02	0.07	0.08	-0.01	-0.08	0.46	-0.54
	Calcium (mg)	5.11	1.87	3.24	-5.89	-1.40	-4.49	26.70	23.32	3.38
	Iron (mg)	-0.29	0.05	-0.34	-0.56	-0.00	-0.56	-0.46	0.37	-0.83
	Zinc (mg)	0.16	-0.02	0.18	0.06	-0.06	0.12	-0.14	0.25	-0.39
	Selenium (μg)	1.73	-0.25	1.98	2.10	-0.37	2.47	-1.67	0.49	-2.16

Notes: Vitamin A is measured by Retinol Equivalent.

Table S.9 A comparison of dietary indicator between urban rich family and rural poor family

	Family type	Urban rich (n=94)		Rural poor (n=16)	
		Mean	S.D.	Mean	S.D.
Food consumption	Cereals (g)	258.99	135.62	274.94	136.94
	Vegetables (g)	178.09	128.67	160.29	124.05
	Meat and poultry (g)	65.68	67.32	53.23	36.02
	Aquatic products (g)	11.54	24.59	10.83	18.71
	Eggs (g)	23.30*	33.04	11.46	19.05
	Dairy products (g)	0.89*	8.60	43.33	83.22
	Fruits (g)	44.89	81.32	55.94	85.35
Nutrition intake	Calorie (kilocalorie)	1188.37*	509.54	941.14	455.77
	Carbohydrate (g)	203.19	93.36	165.19	80.60
	Fat (g)	26.29**	19.66	18.12	12.17
	Protein (g)	41.03	18.03	36.01	16.28
	Vitamin A (μg)	335.00	380.39	296.76	238.65
	Vitamin B (mg)	10.13	5.10	9.37	4.31
	Vitamin C (mg)	47.37	47.50	43.75	27.82
	Vitamin E (mg)	7.10	5.26	6.72	5.60
	Calcium (mg)	242.67	216.25	251.24	144.46
	Iron (mg)	12.48	7.35	11.81	6.37
	Zinc (mg)	6.80	3.00	5.79	2.97
	Selenium (μg)	25.87**	16.37	19.08	9.19

Notes: *, **, *** refer statistically significant at 10%, 5%, and 1%. Vitamin A is measured by Retinol Equivalent. Comparison is conducted between urban rich family and rural poor family.

Formatted: Normal

Table S.10 ATT of single-parent children in rural rich families and urban poor families.

	Children	Rural rich family ATT	Rural rich family Income Effect	Rural rich family Compensation effect	Urban poor family ATT	Urban poor family Income Effect	Urban poor family Compensation effect
Food consumption	Cereals (g)	-52.43	2.48	-54.91	9.50	6.01	3.49
	Vegetables (g)	68.87**	4.68	64.19	5.53	-0.27	5.80
	Meat and poultry (g)	-26.80	-7.00	-19.80	13.89	11.24	2.65
	Aquatic products (g)	11.14	0.38	10.76	-0.40	-1.78	1.38
	Eggs (g)	9.25	5.71	3.54	4.16	-2.03	6.19
	Dairy products (g)	-53.79***	-2.37	-51.42	-1.43	-2.16	0.73
	Fruits (g)	-26.44	0.74	-27.18	8.54	-0.01	8.55
Nutrition intake	Calorie (kilocalorie)	-442.30***	-11.37	-430.93	153.05*	82.25	70.80
	Carbohydrate (g)	-65.87**	2.55	-68.42	26.75*	9.15	17.60
	Fat (g)	-15.08***	-2.31	-12.77	3.37	4.06	-0.69
	Protein (g)	-10.76*	-0.13	-10.63	3.27	1.53	1.74
	Vitamin A (μg)	30.71	-22.33	53.04	49.43	-18.00	67.43
	Vitamin B (mg)	-1.64	-0.09	-1.55	0.34	0.40	-0.06
	Vitamin C (mg)	6.32	1.51	4.81	7.52	-1.16	8.68
	Vitamin E (mg)	0.18	-0.09	0.27	0.41	-0.58	0.99
	Calcium (mg)	-54.35	-6.60	-47.75	10.68	-13.13	23.81
	Iron (mg)	-3.68**	-0.28	-3.40	0.48	0.13	0.35
	Zinc (mg)	-2.63***	-0.12	-2.51	0.98*	0.50	0.48
	Selenium (μg)	-18.68***	-2.04	-16.64	3.35	-0.42	3.77

Notes: *, **, *** refer statistically significant at 10%, 5%, and 1%. Vitamin A is measured by Retinol Equivalent. Comparison is based on values in initial period.

Formatted: Font: Times New Roman, Font color: Red

Table S.~~10~~ 11 A comparison of food consumption and nutrition intake between single-mother and single-father

	Dietary indicator	Single-mother (n=93)			Single-father (n=38)		
		Dual-	Single-	Diff.	Dual-	Single-	Diff.
Food consumption	Cereals (g)	442.53	399.73	-42.80	420.80	434.43	13.63
	Vegetables (g)	322.77	269.53	-53.24**	276.35	280.86	4.51
	Meat and poultry (g)	82.27	71.00	-11.27	56.54	72.09	15.55
	Aquatic products (g)	19.50	10.85	-8.65*	9.65	10.16	0.51
	Eggs (g)	18.95	23.61	4.66	10.48	14.88	4.40
	Dairy products (g)	1.79	2.50	0.71	3.68	0.00	-3.68
	Fruits (g)	20.65	48.89	28.24**	9.49	48.91	39.42***
Nutrition intake	Calorie (kilocalorie)	1702.33	1536.73	-165.60*	1676.96	1686.69	9.73
	Carbohydrate (g)	312.41	279.67	-32.74*	313.36	303.89	-9.47
	Fat (g)	27.85	27.37	-0.48	23.20	27.76	4.56
	Protein (g)	60.07	51.26	-8.81**	52.37	51.50	-0.87
	Vitamin A (μg)	512.90	407.13	-105.77	321.83	368.07	46.24
	Vitamin B (mg)	15.24	12.53	-2.71**	13.16	14.94	1.78*
	Vitamin C (mg)	76.77	72.78	-3.99	63.54	72.15	8.61
	Vitamin E (mg)	9.00	8.31	-0.69	7.27	7.31	0.04
	Calcium (mg)	317.71	301.67	-16.04	286.59	271.36	-15.23
	Iron (mg)	19.73	16.59	-3.14**	16.39	16.76	0.37
	Zinc (mg)	10.01	8.77	-1.24**	9.50	9.64	0.14
	Selenium (μg)	36.64	34.42	-2.22	27.71	29.82	2.11

Notes: *, **, *** refer statistically significant at 10%, 5%, and 1%. Vitamin A is measured by Retinol Equivalent. Comparison is based on values in initial period.
